# Comparative transcriptomics and gene expression divergence associated with homoploid hybrid speciation in *Argyranthemum*

**DOI:** 10.1093/g3journal/jkad158

**Published:** 2023-07-21

**Authors:** Oliver W White, Alfredo Reyes-Betancort, Mark A Carine, Mark A Chapman

**Affiliations:** Algae, Fungi and Plants Division, Department of Life Sciences, The Natural History Museum, Cromwell Road, London SW7 5BD, UK; Jardín de Aclimatación de La Oratava (ICIA), Puerto de la Cruz 38300, Spain; Algae, Fungi and Plants Division, Department of Life Sciences, The Natural History Museum, Cromwell Road, London SW7 5BD, UK; Biological Sciences, University of Southampton, Southampton SO17 1BJ, UK

**Keywords:** transcriptomics, homoploid hybrid speciation, *Argyranthemum*, hybridization, speciation, Plant Genetics and Genomics

## Abstract

Ecological isolation is increasingly thought to play an important role in speciation, especially for the origin and reproductive isolation of homoploid hybrid species. However, the extent to which divergent and/or transgressive gene expression changes are involved in speciation is not well studied. In this study, we employ comparative transcriptomics to investigate gene expression changes associated with the origin and evolution of two homoploid hybrid plant species, *Argyranthemum sundingii* and *A. lemsii* (Asteraceae). As there is no standard methodology for comparative transcriptomics, we examined five different pipelines for data assembly and analysing gene expression across the four species (two hybrid and two parental). We note biases and problems with all pipelines, and the approach used affected the biological interpretation of the data. Using the approach that we found to be optimal, we identify transcripts showing DE between the parental taxa and between the homoploid hybrid species and their parents; in several cases, putative functions of these DE transcripts have a plausible role in ecological adaptation and could be the cause or consequence of ecological speciation. Although independently derived, the homoploid hybrid species have converged on similar expression phenotypes, likely due to adaptation to similar habitats.

## Introduction

Homoploid hybrid speciation (HHS) is the origin of a new species by hybridization without a change in chromosome number (Rieseberg 1997; Soltis and Soltis 2009). Although this phenomenon is traditionally thought to be a rare occurrence in nature, the number of putative homoploid hybrid species documented is increasing, indicating it may be a more widespread phenomenon than we currently think (Mavárez and Linares 2008; Nolte and Tautz 2010; Abbott *et al*. 2013). The accumulation of reproductive barriers between a hybrid lineage and its parents as a result of hybridization is a key criterion of HHS (Schumer *et al*. 2014), but has been proven experimentally in only a handful of cases, i.e. sunflowers (*Helianthus* L.; Rieseberg *et al*. 2003) and passion vine butterflies (*Heliconius* Kluk; Salazar *et al*. 2010). Two mechanisms, chromosomal recombination and ecological isolation, are thought to contribute to the reproductive isolation between a novel hybrid and its parents (Gross and Rieseberg 2005), and it is likely that both act in concert (Rieseberg 1997; Buerkle *et al*. 2000). Indeed, a recombinant genome in a hybrid containing novel combinations of parental alleles may evolve due to selection for ecological divergence (Rieseberg *et al*. 2003). Computer simulations indicate that HHS is unlikely in the absence of niche divergence (Buerkle *et al*. 2000), and this is backed up by empirical data showing that the majority of putative homoploid hybrid species occupy novel ecological habitats that are either intermediate (Brochmann *et al*. 2000; White *et al*. 2018) or extreme (Rieseberg *et al*. 2003; Howarth and Baum 2005; Schwarz *et al*. 2007) with respect to their parents (Gross and Rieseberg 2005; Kadereit 2015).

The underlying genomic or transcriptomic bases of HHS are not well understood. The combination of divergent genomes by hybridization results in dramatic changes in gene expression (Hegarty *et al*. 2008), which could, in theory, lead to ecological displacement, isolation, and origin of a homoploid hybrid species (Lai *et al*. 2006; Hegarty *et al*. 2009). Microarray analyses have identified differentially expressed genes associated with the origin of two homoploid hybrid species, *Helianthus deserticola* Heiser (Lai *et al*. 2006) and *Senecio squalidus* L. (Hegarty *et al*. 2009). Comparative transcriptomic analyses across species are ideally suited for the identification of differentially expressed genes, but, with the exceptions of the two case studies above, and a recent study in birds (Papoli Yazdi *et al*. 2022), comparisons of homoploid hybrids and their parents using this approach have received relatively little attention.

*Argyranthemum* Webb (Asteraceae; Anthemideae) is a genus of flowering plants endemic to the Macaronesian archipelagos of the North Atlantic Ocean, including Madeira, the Selvagens, and the Canary Islands (Humphries 1976) and provides a well-documented model for investigating HHS. *Argyranthemum sundingii* Borgen and *A. lemsii* Humphries are homoploid hybrid species, independently derived from crosses between *A. broussonetii* (Pers.) Humphries and subspecies of *A. frutescens* (L.) Sch.Bip. (Supplementary Fig. 1; White *et al*. 2018). Brochmann *et al*. (2000) proposed that *A. sundingii* and *A. lemsii* should be treated as conspecific, however, because they are independently derived and can be distinguished based on leaf area, we treat them as distinct (White *et al*. 2018). In the Anaga Peninsula of Tenerife in the Canary Islands, *A. frutescens* occupies lowland coastal xerophytic habitats whilst *A. broussonetii* is distributed in higher laurel forests (Humphries 1976; Supplementary Fig. 2; Bramwell and Bramwell 2001). Two subspecies of *A. frutescens* have been implicated in the parentage of the homoploid hybrid species based on their geographical distributions: subsp. *frutescens* as a parent of *A. sundingii* and subsp. *succulentum* Humphries as a parent of *A. lemsii*. Although the parental species can be found within approximately 2 km of each other, they are separated by a steep altitudinal and ecological gradient characterized by decreasing temperature and increasing rainfall/humidity as altitude increases. The homoploid hybrid species are found at intermediate altitudes in South (*A. sundingii*) and North-East (*A. lemsii*) facing valleys of the Peninsula, and niche modeling has demonstrated that the hybrid species occupy distinct ecological niches with respect to each other and their parents (White *et al*. 2018). Despite the homoploid hybrid species being independently derived, they have a similar genomic composition, with approximately 80% of the genome derived from *A. broussonetii* and 20% from *A. frutescens* (White *et al*. 2018).

The aim of this paper is to investigate the changes in gene expression associated with HHS in *Argyranthemum*. For non-model species without a reference genome, there are different methods available for the assembly and expression quantification of RNA-seq data. Comparative transcriptomics, i.e. the comparison of gene expression divergence across species, requires bioinformatic approaches which accurately identify orthologous loci across species such that paralogous loci are not misidentified as orthologs, and that sequence divergence between species does not prevent ortholog identification. Since there is no “gold standard” method to achieve this, we trialled a range of pipelines in an attempt to find one that optimizes the amount of data analysed and suffers the least bias.

**Fig. 1.**
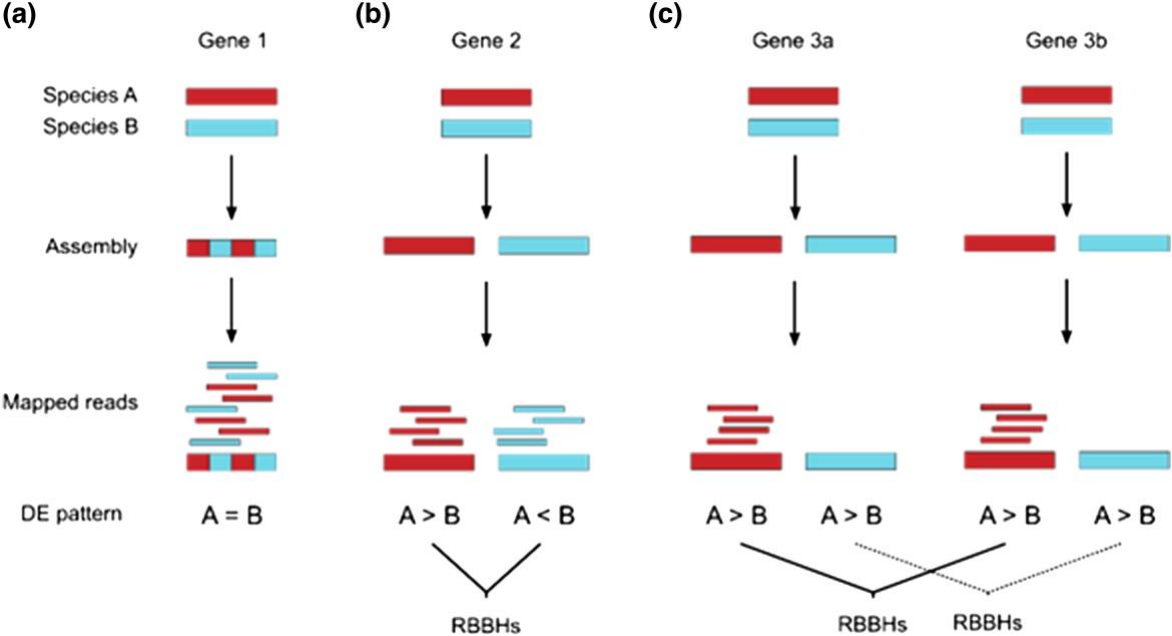
Schematic diagram of correctly and incorrectly identified differential expression scenarios. a) Gene 1 is sufficiently similar between the two species that the reads co-assemble and, because expression is similar in the two species no differential expression (DE) is identified. b) Gene 2 has diverged such that the reads from this gene from each species do not co-assemble. Although each species has a similar expression, there is a false impression of differential expression (DE). Under this scenario, these genes can be identified as reciprocal best blast hits (RBBHs) that are reciprocally DE. c) Genes 3a and 3b are DE paralogs and each paralog does not co-assemble (as in b). Genes 3a and 3b are rightly identified as DE and can be identified as RBBHs that are DE in the same direction.

For studies in which RNA-seq data are assembled from multiple species into a single assembly (e.g. Chapman *et al*. 2013), one assumes that orthologous loci in each taxon are sufficiently similar that they will “co-assemble” (Fig. 1a). However, depending on the extent of genetic divergence between the study species, a subset of orthologous loci may have diverged sufficiently to prevent co-assembly, leading to the separate assembly and potentially false patterns of differential expression (Fig. 1b). These loci would be identifiable as reciprocal best blast hits (RBBHs) that are reciprocally differentially expressed (e.g. species A > B for one locus and species A < B for the other). Paralogous loci that are both genuinely DE would also be identified as RBBHs (Fig. 1c), however, we would expect the pattern of DE to be in the same direction if the paralogs have similar function and selection has caused divergence of gene expression (e.g. A > B for both loci).

One way to avoid the issue of reads not assembling correctly is to assemble species' transcriptomes individually and identify and combine orthologous loci across species, using a clustering algorithm such as CD-HIT (Li and Godzik 2006; Fu *et al*. 2012), which is frequently employed to cluster similar sequences from independently assembled transcriptomes (Hodgins *et al*. 2015; Roberts and Roalson 2017; Ru *et al*. 2018). However, the appropriate clustering threshold will vary based on the genetic divergence between the species being tested, and potentially among genes in the genome due to different rates of evolution (Clark *et al*. 2007), and it therefore can only collapse similar sequences, which may or may not be orthologs. Another possibility is to assemble the data within species and then parse down to only the one-to-one orthologs, therefore enriching for true orthologs (Wright *et al*. 2015; Harrison *et al*. 2015). This will remove loci lowly expressed in one or more samples, and if identifying differential expression is the goal, the most highly differentially expressed loci (e.g. no expression in one taxon) would be removed, negating the experiment. A final possibility includes the use of orthogroups, a set of genes that are descended from the last common ancestor of the species being considered (Emms and Kelly 2015). Orthogroups are often used in comparative genomics (Hodgins *et al*. 2015; Grusz *et al*. 2016; Roberts and Roalson 2017) and their use circumvents some of the disadvantages listed above for the other approaches. However, by definition, orthogroups can also include paralogs which therefore means that “DE loci” are sometimes groups of loci.

Five pipelines for transcriptome assembly and ortholog identification were investigated. After selecting a pipeline that optimized the comparison of expression across taxa, we compared gene expression among the taxa relevant to our examples of HHS within *Argyranthemum*. First, we compare the parental taxa (Table 1A), identifying differentially expressed (DE) loci that may have diverged due to local selection, for example, due to the environment. Second, we examine gene expression in the homoploid hybrid species to determine the proportion of loci with novel expression (transgressive and/or intermediate; Table 1B) or parent-like expression (Table 1C). We hypothesize that differential expression will evolve in loci conferring morphological differences, local adaptation, and/or reproductive isolation between the taxa. Third, we assess the degree to which the independently derived homoploid hybrid species have converged on similar gene expression profiles (Table 1D). We hypothesize that due to the similar environment of the two homoploid hybrid species there will be convergence in gene expression divergence, despite the independent origins. We investigated gene expression using plants grown under common conditions to control for expression differences due to the environment, acknowledging that if any loci confer local adaptation solely because of environmentally induced gene expression differences (i.e. expression plasticity) these will be missed in our analyses.

**Table 1.** Expression phenotypes used to identify transcripts that are DE between the parental species (A), with novel (B) or parent-like (C) expression in the homoploid hybrid species, and DE between the two homoploid hybrid species (D).

Type	Expression phenotype	bro v fru	bro v sun	bro v lem	fru v sun	fru v lem	sun v lem
A	DE between fru and bro	≠	**-**	**-**	**-**	**-**	**-**
B	Novel sun	**-**	≠	**-**	≠	**-**	≠
	Novel lem	**-**	**-**	≠	**-**	≠	≠
	Novel sun + lem	**-**	≠	≠	≠	≠	**=**
C	sun bro-like	≠	**=**	**-**	≠	**-**	**-**
	sun fru-like	≠	≠	**-**	**=**	**-**	**-**
	lem bro-like	≠	**-**	**=**	**-**	≠	**-**
	lem fru-like	≠	**-**	≠	**-**	**=**	**-**
	sun + lem bro-like	≠	**=**	**=**	≠	≠	**=**
	sun + lem fru-like	≠	≠	≠	**=**	**=**	**=**
D	DE between sun and lem	**-**	**-**	**-**	**-**	**-**	≠

Taxa are abbreviated using the first three letters of the species name. Symbols indicate DE (≠), not DE (=) and either pattern (−).

## Materials and methods

### Sampling and RNA extraction

Seed (cypselae) were sampled in 2015 from distinct populations across the species ranges of *A. broussonetii*, *A. frutescens*, *A. sundingii*, and *A. lemsii* (Supplementary Table 1 and Supplementary Fig. 2). Plant material was collected under a permit from the Cabildo de Tenerife, number 18297, and Gobierno de Canarias permit number 2015/939. Germination was enhanced through chipping the seed and applying gibberellic acid overnight at 4°C and then rinsing with sterile water (Francisco-Ortega *et al*. 1994), prior to sowing directly onto a 2:1 mix of compost and perlite in a 5 cm diameter pot with a maximum of two cypselae per pot. Pots were covered with cling-film for the first ten days and bottom-watered daily in an environmentally controlled room (16 h daylength, 60% humidity, 23°C day, 18°C night). To minimize gene expression difference due to morphological differences, plants (six per species) were sampled at the same developmental stage, specifically the expansion of the third true leaf (true leaves are those that develop after the cotyledon leaves). Images of seedlings at the sampling stage are presented in Supplementary Fig. 3. In addition, all plants were sampled between 11 Am and 12 PM to minimize differences due to circadian rhythms. The third true leaf was snap frozen in liquid nitrogen before RNA extraction using RNeasy Plant Mini Kits (QIAGEN, Manchester, UK) following the manufacturer's instructions and utilizing the on-column DNase digestion step (RNase-free DNase, QIAGEN). RNA concentration was evaluated using a Quantus Fluorometer (Promega, Southampton, UK) and quality was checked by agarose gel electrophoresis.

### Sequencing and pre-processing

Two µg of RNA per sample was shipped to Novogene (Hong Kong), where RNA Integrity Number was calculated using an Agilent 2100, and only samples with RIN > 6.0 were used. Paired-end sequences were generated for samples (after poly-A isolation) on an Illumina HiSeqX for 150 cycles. Poor-quality sequence and adapters were removed with Trimmomatic version 0.32 (Bolger *et al*. 2014) with the following parameters: Illumina clip 2, palindrome clip 30, simple clip 10, minimum adapter length 8, leading quality and trailing quality 5, window size 4, required quality 15, and minimum read length of 36. Reads which remained as a pair were used in the downstream analyses.

### *De novo* assembly and annotation

We investigated five pipelines for the assembly and ortholog identification (Supplementary Fig. 4). Assembly was carried out using Trinity version 2.11 (Grabherr *et al*. 2013) because it was one of the most reliable transcriptome assemblers in a recent benchmarking exercise (Hölzer and Marz 2019). Read normalization to k-mer coverage of 30 was carried out prior to assembly. Reads were normalized within species using Trinity, and for the interspecific assembly (detailed below) the normalized reads from the four species were combined and normalized again in the same way.

For transcriptome assembly, transcripts were assembled with minimum k-mer coverage of 2, increasing the stringency for reads to be assembled together. To account for polymorphism between individuals and species, up to 4 nucleotide differences and a 15 bp gap were allowed when assembling transcripts. Whilst varying these parameters could affect the correct assembly of orthologs, altering the number of nucleotide differences and gap sizes made little effect on the overall assembly (Supplementary Table 2), and so we did not attempt to optimize this further. Trinity assembles the reads into isoforms (‘transcripts’ from hereon), and multiple isoforms that are related due to alternative splicing or gene duplication are grouped *in silico* as a Trinity “gene” (Grabherr *et al*. 2013).

Transcripts from the interspecific and species-specific assemblies were annotated separately using a blastn search (Camacho *et al*. 2009) against representative CDS sequences (primary transcript only) from *Helianthus annuus* L. (Badouin *et al*. 2017) and *Arabidopsis thaliana* (L.) Heynh (Lamesch *et al*. 2012) downloaded from Phytozome (https://phytozome.jgi.doe.gov/pz/portal.html; accessed 17/07/2018). An e-value cutoff of 10^−20^ was used and 500 maximum target sequences were retained. The top hit (i.e. that with the lowest e-value) was then selected. If two hits had the same e-value, one was selected at random.

### Quantifying transcript abundance and differential expression (see Supplementary Text: Methods for full details)

Pipeline 1—Transcript abundance for each sample was estimated by mapping filtered (non-normalized) reads to the interspecific assembly (Li and Dewey 2011). Transcript abundance was quantified at the level of Trinity isoforms.

Pipeline 2—CD-HIT-EST (Li and Godzik 2006; Fu *et al*. 2012) was used to identify similar transcripts within the interspecific assembly, the longest was retained, and transcript abundance quantified as for pipeline 1.

Pipeline 3—The four species-specific assemblies were combined, and CD-HIT-EST was used in the same way as pipeline 2. Transcript abundance was then quantified as for pipelines 1 and 2.

Pipeline 4—For each species-specific assembly, peptide open reading frames were predicted and the longest ORF was retained. CD-HIT was used to cluster peptide sequences within each species using a similarity threshold of 0.995. One-to-one orthologs between species were identified and collapsed. Gene expression was quantified using a reference transcriptome comprising a concatenation of the four species-specific transcriptome assemblies and transcript expression was quantified at the level of the one-to-one orthologs identified.

Pipeline 5—We carried out the same steps as for pipeline 4, but we also included representative peptide sequences (primary transcript only) from five reference taxa, *Helianthus annuus* (Badouin *et al*. 2017), *Lactuca sativa* L. (Reyes-Chin-Wo *et al*. 2017), *Solanum lycopersicum* L. (The Tomato Genome Consortium 2012), *Mimulus guttatus* DC. (Hellsten *et al*. 2013), and *Arabidopsis thaliana* (Lamesch *et al*. 2012) in our OrthoFinder analysis to improve orthogroup inference. We did not restrict this analysis to the one-to-one orthologs. A significant number of orthogroups appeared to contain multiple gene paralogs, hence we carried out additional filtering to remove these (see Supplementary Text: Methods). After this, expression was quantified in the same way as pipeline 4, again quantifying transcript expression at the level of the orthogroup.

Following transcript quantification, an expression matrix with counts and TMM-normalized counts was built for each pipeline using Trinity. Differentially expressed (DE) transcripts, orthologs or orthogroups (hereafter, simply loci) were identified in pairwise comparisons amongst species using edgeR (Robinson *et al*. 2009; McCarthy *et al*. 2012) in R (R Core team 2020) within Trinity. The cutoff for differentially expressed transcripts was a *P* value < 0.05 after false discovery rate correction (Benjamini and Hochberg 1995). No fold-change cutoff was used as there is no reasons to assume that a greater fold change is more important that a lower fold change.

Note that we did not remove lowly expressed loci/orthogroups for any pipeline following the recommendation in the Trinity documentation since this removal could result in the loss of biologically relevant transcripts. Nevertheless, to examine the effect of lowly expressed transcript removal, we carried out a parallel analysis of pipeline 5 but after removing transcripts with low expression (<1 TPM in all samples). This made minor differences to the results with ca. 80% of DE transcripts and over-represented GO terms found in both analyses (Supplementary Fig. 5).

### Pipeline comparison (see Supplementary Text: Methods)

To examine whether pipeline choice could result in fundamentally different biological conclusions, we identified Gene Ontology (GO) terms that were over-represented in the lists of loci DE between the parents using TopGO (Alexa and Rahnenfuhrer 2021) with significantly enriched terms identified using a *P* value < 0.01 with the weight01 algorithm. To select a pipeline for further analysis, we aimed to determine the extent to which true orthologs were not correctly assembled in each pipeline. If this were the case, we would expect to find that DE transcripts in that pipeline are more likely than expected by chance to have a reciprocal best blast hit (RBBH) which is also DE (i.e. Figure 1b). We therefore quantified for each pipeline the number of RBBHs which were both DE, one was DE, or neither was DE, and used χ^2^ tests to determine if there was deviation from that expected by chance, and used a φ correlation to estimate skew in the χ^2^ test.

This first test does not inform us whether the skew is due to orthologs not co-assembling (Fig. 1b) or DE paralogs not co-assembling (Fig. 1c). To differentiate these we further identified, within the RBBHs where both of the pair are DE, those which were reciprocally DE (i.e. Figure 1b) and those which were DE in the same direction (i.e. Figure 1c). We reason that if the pipeline had reduced the problem of orthologs not co-assembling then we would expect less reciprocally DE transcripts than expected by chance (compared to the direction of DE in all RBBHs).

From these two tests, the pipeline with the least evidence of having incorrectly assembled/grouped true orthologs, whilst also optimizing the number of loci available for the expression analyses, was used for further analyses of the data to examine HHS.

### Gene expression divergence and HHS

For the selected pipeline (see Results), we compared expression across all four species, identified DE loci, and identified over-represented GO terms as above. We also identified enriched KEGG pathways using ClusterProfiler (Wu *et al*. 2021) with a *P* value threshold of 0.05. In addition to the inter-parental DE analysis (Table 1A), we identified novel expression phenotypes in the hybrid species (Table 1B), parent-like expression in the homoploid hybrid species (Table 1C), and finally DE between the two homoploid hybrid species (Table 1D).

Finally, to assess the degree to which the independently derived homoploid hybrid species have converged on similar gene expression we generated a sample correlation matrix and carried out principal component analysis using the Trinity script *PtR* with counts per million and log_2_transformation.

## Results

### RNA-seq processing and assembly

RNA-seq from six individuals of each species produced a total of 674 M raw paired reads with an average of 28.1 M (± 0.9 M [SE]) per sample. Filtering to remove poor-quality sequences and short reads removed approximately 5.21% of reads (Supplementary Table 3). Normalization within species retained on average 9.52 M reads (5.96% of the input) and when the normalized reads were combined and re-normalized (for the interspecific assembly) this retained 20.59 M of the 38.06 M reads (54.10%; Supplementary Table 4).

Assemblies were generated for all species together (“interspecific assembly”) or on a species-by-species basis (“species-specific assemblies”). The interspecific assembly had ca. 459 K genes and 794 K transcripts, with an average contig length of 683 bp and N50 of 994 bp (Supplementary Table 5). The species-specific assemblies (White *et al.* 2023) resulted in an average of ca. 206 K genes and 337 K transcripts per species (range 192 K to 222 and 320 to 358 K, respectively), with an average contig length of 779 bp and N50 of 1207 bp (Supplementary Table 5). Between 70.4% (±1.3% [SE] for *A. frutescens*) and 74.6% (±0.6% for *A. broussonetii*) of the reads successfully mapped back to each species' reference assembly.

Five pipelines for the estimation of expression across species were implemented (Supplementary Fig. 4 and Supplementary Text: Methods). In pipeline 1, the interspecific assembly (see above) was used as a reference sequence to quantify expression. In pipeline 2, putative orthologous transcripts within the interspecific assembly were collapsed with CD-HIT-EST which resulted in 556 K transcripts. For pipeline 3, the species-specific assemblies were combined and similar transcripts were collapsed as above which resulted in 667 K transcripts. In pipeline 4, running OrthoFinder with only transcripts from *Argyranthemum* identified 57,660 orthogroups, only 3,774 of which (6.5%) were retained for the analysis because they are one-to-one orthologs. Removing 93.5% of loci is clearly suboptimal, and those removed will be enriched for those which are DE (i.e. the ones we are attempting to identify). For pipeline 5, a total of 49,650 orthogroups were identified. Of these, 5,274 were removed as likely paralog-containing (due to non-monophyly of transcripts within genera). This resulted in 44,376 orthogroups, of which 79.4% were comprised of 10 or less transcripts and 60.5% five or less transcripts (Supplementary Fig. 6). 37,256 were comprised of transcripts from *Argyranthemum* (Table 2). Assembly, clustering and annotation details are presented in Table 2.

**Table 2.** Summary statistics of the pipelines compared in the present study.

	Pipeline 1	Pipeline 2	Pipeline 3	Pipeline 4	Pipeline 5
*N* transcripts in assembly	794,264	794,264	1,346,940	1,346,940	1,346,940
*N* transcripts after collapsing*^a^*	-	556,146	667,289	321,870	321,870
*N* orthologs or orthogroups	-	-	-	3775	37,256
*N* DE between bro and fru	16,834	13,489	28,308	166	2786
% DE between bro and fru	2.12%	2.43%	4.24%	4.40%	7.48%
Annotated number in query list*^b^*	4892	3957	6654	105	1045
Annotated number in background*^c^*	12,125	11,836	12,031	2307	8604
Significant GO terms	2298	1635	2777	18	243
χ^2^	611.49	529.38	570.30	6.50	14.02
χ^2^ *P* value	5.3× 10^−135^	3.8× 10^−117^	4.8× 10^−126^	0.0108	0.0002
φ	0.208	0.226	0.200	0.125	0.053
Number (%) reciprocal DE*^d^*	629 (56.6)	365 (40.3)	1,126 (49.0)	1 (25.0)	18 (19.4)
Number (%) same direction DE*^d^*	483 (43.4)	540 (59.7)	1,170 (51.0)	3 (75.0)	75 (80.6)

Number of transcripts after collapsing by CD-HIT-EST (0.95 similarity) or CD-HIT (0.995 similarity) for pipelines 2–3 and 4–5, respectively.

Number of genes annotated with GO terms that are differentially expressed between bro and fru.

Number of genes annotated with GO terms across all found in bro and fru.

Based on the subset of RBBHs where both members are DE.

### Pipeline comparison and choice for downstream analyses

Differential expression between the parental species was compared for the five pipelines. The number (and percentage) of DE loci for pipelines 1, 2, and 3 were 16,834 (2.12%), 13,489 (2.43%), and 28,308 (4.24%), respectively. For pipelines 4 and 5 the equivalent numbers were 166 (4.40% of single-copy orthologs) and 2,786 (7.48% of orthogroups). Between 18 (pipeline 4) and 2,777 (pipeline 3) significant GO terms were resolved for the DE loci (Table 2).

A total of 15 GO terms were over-represented in all five analyses (Supplementary Fig. 7), which included the GO term *response to light stimulus* (GO:0009416). Excluding pipeline 4, a total of 186 GO terms were shared between the pipelines, which included *response to water deprivation* (GO:0009414), *response to response to salt stress* (GO:0009651) and *response to cold* (GO:0009409). Pipelines 1, 2, and 3 each have relatively distinct GO terms with 8, 39, and 21.4% unique respectively (Supplementary Fig. 7). In contrast, pipelines 4 and 5 are much less distinct with less than 1% of the GO terms being pipeline-specific.

In all pipelines, χ^2^ tests found that RBBHs where both members are DE occurred more often than expected by chance (all *P* < 0.05; Table 2). This is presumably, at least in part, due to sequence divergence which could prevent some orthologous loci co-assembling (see Supplementary Text: Results). The skew in the χ^2^ test, assessed by a φ correlation (where 0 is no skew and 1 is strong skew; Table 2), was greatest for pipelines 1–3 (all φ ≥ 0.2), intermediate for pipeline 4 (0.125) and least for pipeline 5 (0.053).

We also suggest above that some skew might be expected if two (relatively divergent and therefore non-co-assembling) gene paralogs are genuinely DE (Fig. 1c); as might be expected if the paralogs perform a similar function. To investigate this, we examined the direction of DE (i.e. bro > fru or bro < fru) in all pairs of RBBHs where both members are DE.

Excluding pipeline 4 (see below), very similar number of bro > fru and bro < fru transcripts were present, resulting in an almost 50:50 expectation of reciprocally and same direction DE RBBHs (i.e. orthologs not co-assembling vs paralogs not co-assembling, respectively). Our results (see Supplementary Text: Results) matched this expectation for pipelines 1–3, whereas for pipeline 5 the observed percentage was only 19.4% (Table 2). This suggests that for pipeline 5 we had substantially reduced the number of putative non-co-assembling orthologs. For pipeline 4, for only four pairs of RBBHs (eight loci) were both DE, with a skew in the number of bro > fru vs bro < fru transcripts present (7 vs 1), resulting in an estimation of 22% of pairs being reciprocally DE, which matched the observed result (one out of the four; i.e. 25%).

Considering the evidence above we employed pipeline 5 for further downstream analyses as it appears to reduce the problem of orthologous loci not co-assembling (seen in pipelines 1–3) and does not remove a large proportion of loci prior to analysis (seen in pipeline 4).

### Global gene expression variation between the parents and hybrid species

**Fig. 2.**
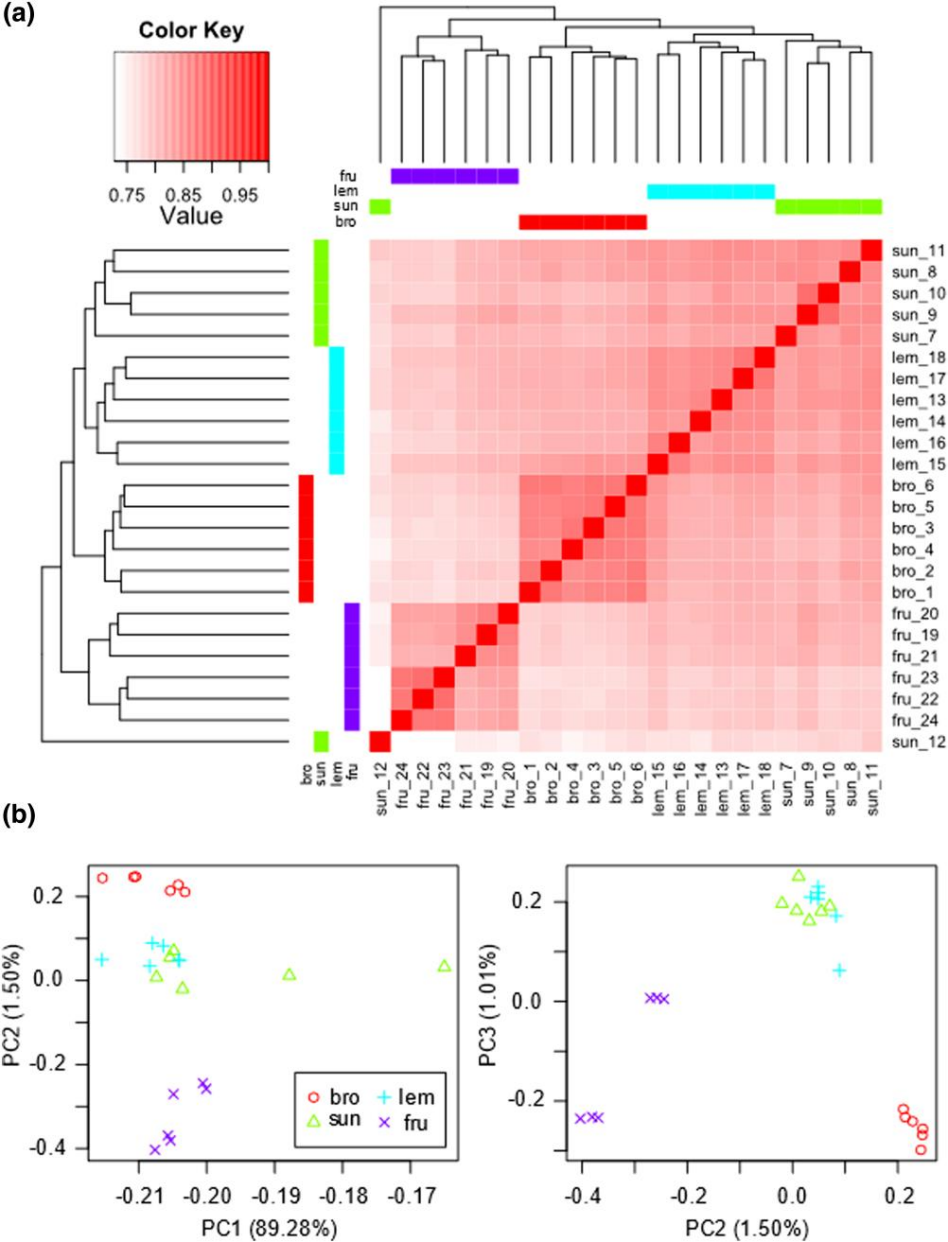
Sample correlation matrix (a) and principal components analysis (b) of expression. The sample correlation matrix shows Pearson's correlation in expression between samples and the dendrograms above and to the left show similarity/dissimilarities between samples. In b, the first, second, and third axes of the principal component analysis are shown.

Across the whole transcriptome, there was a clear distinction in gene expression between the parental species, and between the two subspecies of *A. frutescens* (samples 19–21 vs samples 22–24; Fig. 2). One sample (*A. sundingii* 12; Fig. 2a) appears to be an outlier, but overall it appears that the homoploid hybrids have converged on similar expression profiles, are distinct from the parental progenitors, and are more similar in expression phenotype to *A. broussonetii* than to *A. frutescens* (Fig. 2b).

### Extensive expression divergence in genes putatively involved in local adaptation

Across comparisons, the greatest number of DE loci (i.e. orthogroups) and enriched GO terms was found between the parents, with 2,786 and 243, respectively (Table 3). GO terms were associated with a wide variety of biological processes (*n* = 133), cellular component (32) and molecular functions (78; Supplementary Table 6). The three most enriched GO terms were biological process (GO:0008150; an umbrella term for any biological process), response to water deprivation (GO:0009414), and protein phosphorylation (GO:0006468). Other GO terms significantly enriched in the orthogroups DE between the parents include response to temperature stimulus (GO:0009266), and response to stress (GO:0009651) which could plausibly indicate divergence of genes involved in local adaptation. A single KEGG pathway was enriched, Biosynthesis of amino acids (Supplementary Table 7a).

**Table 3.** Number of differentially expressed (DE) loci, the number with blast hits in *Arabidopsis thaliana*, the number with annotated hits in *A. thaliana*, and the number of significantly enriched gene ontology (GO) terms for each expression phenotype.

Type	Expression phenotype	DE	Blast hit	Annotated	GO terms
A	Between fru and bro	2,786	1,275	45.76%	243
B	Novel sun	14	6	42.86%	8
	Novel lem	11	3	27.27%	17
	Novel sun + lem	33	11	33.33%	34
C	sun bro-like	482	209	43.36%	47
	sun fru-like	464	187	40.30%	49
	lem bro-like	657	306	46.58%	79
	lem fru-like	407	173	42.51%	55
	sun + lem bro-like	261	118	45.21%	55
	sun + lem fru-like	198	79	39.90%	57
D	Between sun and lem	129	48	37.21%	48

Full details are given in Supplementary Tables 12–17.

Relatively few loci exhibited patterns of novel expression in one or both homoploid hybrid species, with only 58 loci expressed in a novel manner in *A. sundingii*, *A. lemsii* or both (Table 3). With one exception, all loci with novel expression phenotypes in one or both of the homoploid hybrid species exhibited a transgressive expression pattern, either upregulated or downregulated with respect to the parents. Eight, 17 and 34 GO terms were enriched for novel expression in *A. sundingii*, *A. lemsii* and both homoploid hybrid species, respectively (Supplementary Table 8–10). The KEGG pathway fatty acid biosynthesis was enriched in both hybrid species, biotin metabolism, flavonoid biosynthesis and N-Glycan biosynthesis were enriched in *A. sundingii*, and carbon fixation was enriched in *A. lemsii*. (Supplementary Table 7).

In *A. sundingii*, 482 loci were *A. broussonetii*-like and 464 were *A. frutescens*-like in their expression. In *A. lemsii*, 657 loci were *A. broussonetii*-like and 407 were *A. frutescens*-like in their expression. For the hybrid species together, there were 261 loci with *A. broussonetii*-like expression and 198 loci with *A. frutescens*-like expression (Table 3). For a locus to be “parental-like” in terms of expression in one or both hybrid species, we focussed on only those loci also DE between the parental species, therefore the GO analysis would be enriched for terms also found in the “DE between the parents’ analysis and hence was not performed. A total of 129 loci were DE between *A. sundingii* and *A. lemsii* (Table 3), and these were significantly enriched for 48 GO terms, including chloroplast organization (GO:0009658) and glucose catabolic process (GO:0006007) (Supplementary Table 11) and KEGG pathways involved in amino acid metabolism, and biosynthesis of fatty acids, biotin, and flavonoids (Supplementary Table 7d).

## Discussion

In this study, we compared a range of pipelines for de novo comparative gene expression analyses (i.e. comparing gene expression across species) between four *Argyranthemum* species. A method based on the identification of orthogroups was employed and differentially expressed (DE) loci were identified with the goal of identifying loci potentially associated with the origin of the homoploid hybrid species. We show that several genes that are DE between the parental species are putatively involved in local adaptation based on Gene Ontology, and that the hybrid species contain a combination of parental-like gene expression and novel (transgressive) expression. Adaptation to a novel environmental niche may be a prerequisite for homoploid hybrid species formation (Lai *et al*. 2006; Schwarz *et al*. 2007; White *et al*. 2018) and our data demonstrate that this is also detectable at the level of the transcriptome.

### Assessment of assembly pipelines

The assembly and analysis pipeline used affects the biological interpretation of the data, in our case assessed through identifying over-represented GO terms. We therefore show that it is important that a pipeline is selected based on the characteristics of the data available.

Solely limiting analyses to the one-to-one orthologs is inappropriate for studying DE because weakly expressed and non-expressed genes will necessarily be removed from the analysis, thus removing the loci of greatest interest. In our pipeline where only the one-to-one orthologs were retained (pipeline 4) ca. 93% of orthogroups were removed. In other studies where only one-to-one orthologs were retained, e.g. Entelegyne spiders (3,345 genes; Carlson and Hedin 2017) and New World lupins (6,013 genes; Nevado *et al*. 2016), the goal was phylotranscriptomics, therefore, removing DE loci was not necessarily of significance to the main goal.

Of the other four pipelines, the one we selected maximizes the proportion of data included in the analysis whilst filtering out orthogroups with the strongest evidence for being comprised of paralogous sequences. Compared to studies based on a single interspecific assembly (pipeline 1), we expect that our results are less likely to recover false patterns of differential expression (Fig. 1). We cannot say that we have definitively removed the problem of orthologs not co-assembling, and it is clear we still have some skew in the data (i.e. DE loci tend to have a best blast hit that is also DE more than chance). However, we might realistically expect this occurrence if, for example, two gene paralogs do not co-assemble, yet are genuinely DE (Fig. 1c). Our supplementary analysis, determining the direction of DE in the RBBHs, does further indicate however that we have reduced the proportion of putative orthologs not co-assembling. It is important to note that biological differences between the species being analyzed (in terms of genetic divergence, gene loss/gain, introgression, and incomplete lineage sorting) would affect the settings to use in future analyses, and it is likely that different pipelines would work better in other study systems.

### DE between the parents and novel expression in the hybrid species

Differential expression was greatest between the parental species, with 2,786 DE orthogroups (7.48% of orthogroups), from which 243 GO terms were significantly over-represented. The parental species occupy the altitudinal extremes of the Anaga peninsula of Tenerife (<200 m *A. frutescens*; >500 m *A. broussonetii*) connected by steep ecological gradients of temperature, rainfall, and humidity (Brochmann *et al*. 2000; Fjellheim *et al*. 2009). Temperature was identified as an important contributor to the distribution of the parental species using ecological niche modeling (White *et al*. 2018), and the GO term *response to temperature* (GO:0009266) was identified in our analysis.

Patterns of novel gene expression were identified in the homoploid hybrid species, with 58 orthogroups expressed in a novel manner in one or both homoploid hybrid species, and except for a single orthogroup, a transgressive pattern of expression was identified (i.e. significantly higher or lower expression than in both parental species). Presumably, the bias in transgressive novel to intermediate novel gene expression comes from the difficulties in identifying the latter due to gene expression variation typically being noisy. This small proportion of DE loci in the hybrid species compared with the parental species could be because the hybrid species originated only 2–4 Mya (White *et al*. 2018), hence, there is comparatively little evolutionary time for gene expression divergence to evolve. The over-represented GO terms do not provide obvious links to adaptation to the non-parental environment, although terms related to root development, flavonoids and auxin homeostasis were over-represented.

As far as we are aware, only two studies in plants have examined gene expression in homoploid hybrid species and their parents, both of which were based on microarrays (Lai *et al*. 2006; Hegarty *et al*. 2009). Genetic divergence between the parents could also give rise to biased expression patterns in microarrays not dissimilar to those we attempt to minimize here. Nevertheless, and similar to our results, in both studies, loci putatively involved in ecological adaptation were present in the lists of loci with transgressive expression phenotypes in the homoploid hybrid species. Together, an overrepresentation of GO terms related to adaptation in the loci DE between homoploid hybrids and the parents supports the hypothesis that ecological divergence is important in the origin of homoploid hybrid species (Lai *et al*. 2006; Hegarty *et al*. 2009), and we show that in some cases this appears to result from the evolution of novel transgressive gene expression.

### Expression differences between the independently originated hybrid species

A small number of differences in gene expression between the two homoploid hybrid species were identified in our study (129 DE loci, considerably less than the 2,786 between the parents). It has recently been shown that the two hybrid species occupy similar but distinct ecological niches in Tenerife, primarily differing in terms of the northern trade winds (White *et al*. 2018). Over-represented GO terms in the loci DE between *A. sundingii* and *A. lemsii* included response to stress (GO:0006950).

Overall, based on the relative paucity of loci DE between the two hybrid species, the shared patterns of novel gene expression, and the intermediate and overlapping gene expression patterns of the two hybrid species, it seems that despite the species originating independently (White *et al*. 2018), the expression phenotypes have converged (see also Fig. 2). Whilst there are other examples of independent origins of homoploid hybrids from the same parental species (e.g. *Helianthus* sunflowers; Rieseberg 1991), ours is the only study to examine convergence of gene expression. Rieseberg *et al*. (1996) recreated three artificial homoploid hybrid sunflowers and demonstrated that their genomic composition, in terms of parental markers retained or lost, was remarkably similar across the three lineages and to the ancient hybrid species. This suggests that independently derived lineages can converge on similar genetic combinations (potentially due to only some genomic combinations being fit), which could, in part, explain the convergence of gene expression we observed.

### Parental inheritance of gene expression in the hybrid species

Previously, we demonstrated that the genomic make-up of the two homoploid hybrid species is not intermediate with respect to the parental species; instead, the hybrid species retain a greater contribution of *A. broussonetii* loci relative to *A. frutescens* (approximately 80:20 for *A. broussonetii* and *A. frutescens* respectively based on STRUCTURE analyses; White *et al*. 2018). However, for gene expression there was a less obvious bias. After combining the species-specific parent-like and the shared parental-like DE loci, for *A. sundingii*, a slight bias towards *A. broussonetii*-like (743) vs *A. frutescens*-like (662) expression was evident (ca. 53:47). For *A. lemsii*, there was a greater bias towards *A. broussonetii*-like expression (918), relative to *A. frutescens*-like (605) expression (ca. 60:40). Overall, it seems that gene expression is slightly biased towards the *A. broussonetii*-like expression phenotype, but not to the extent to which the genomic make up is biased towards loci inherited from *A. broussonetii*.

In one of the two other studies of gene expression in homoploid hybrid species, Lai *et al.* (2006) investigated gene expression divergence between *Helianthus deserticola* and its parental species *H. annuus* and *H. petiolaris*. In this, 2.0%, 3.3%, and 5.8% of 2,897 genes exhibited transgressive, *annuus*-like, and *petiolaris*-like expression, respectively. This therefore shows the same qualitative pattern to our analysis in which novel expression was comparatively rare, and parental-like expression was biased.

### The genomic bases of species differences

Although our methodology maximized the proportion of data being analysed, a relatively small proportion of loci exhibited DE between any pairs of taxa. The four species are all closely related, therefore, it might be that insufficient evolutionary time has passed for more significant expression changes. In a study comparing expression divergence between two closely related species of *Senecio,* also separated by altitude and estimated to have diverged only ca. 153 Kya, only 0.5% of loci were DE between the high and low altitude species (Chapman *et al*. 2013).

We also note that our experimental design does not account for expression plasticity that may underlie adaptive divergence (for example, changes in gene expression associated with the environment) or maternal effects. Further, our analysis was based on transcriptomes from seedling leaf tissue; hence any differential expression in other tissues (e.g. adaptation to different soil would likely be observed in the roots) would be missed.

It is also likely that ecological adaptation and speciation may be conferred to a certain degree by sequence differences as opposed to expression differences. Since our chosen pipeline groups transcripts across species, we cannot generate haplotype sequences from the individuals or species, hence sequences cannot be compared to identify loci exhibiting the hallmarks of divergent selection.

## Conclusions

With two hybrid species independently derived from the same parental species and a likely role for ecological speciation in each case, *Argyranthemum* is a valuable model for studying homoploid hybrid speciation. In this paper, we aimed to test the hypotheses that that DE in the hybrid complex will evolve in loci conferring morphological differences, local adaptation, and/or reproductive isolation between the taxa and that due to the similar environment of the two homoploid hybrid species there will be convergence in gene expression divergence, despite the independent origins. We identified DE loci between pairs of taxa with plausible links between putative functions of the DE genes and the ecological divergence of the taxa. Furthermore, although independently derived, the homoploid hybrid species appear to have converged on similar expression phenotypes, potentially a consequence of adaptation to similar habitats in the Anaga peninsula that are intermediate between the parents, although this could also be evidence that only certain genomic combinations are fit.

The analysis of *Argyranthemum* clearly highlights how the analysis methodology impacts the biological interpretation. Future work could test this hypothesis with simulated data or a model system. However, we assume there will not be a one size fits all approach, and the appropriate pipeline is likely to differ between study systems.

Building upon this study, future work into the genetic basis of ecological divergence and speciation should investigate gene expression in situ, using reciprocal transplant material and could be expanded to sample multiple tissues. In addition, long-read sequencing technology can generate full-length transcripts, circumventing some problems with the initial assembly and allowing researchers to investigate the role of alternative splice variants (Cho *et al*. 2014). Finally, a more in-depth understanding of speciation and divergence would benefit from investigating sequence-based differences between taxa, and the parental pattern of inheritance.

Comparative transcriptomics across species offers a unique opportunity to investigate an important aspect of divergence associated with speciation. It is gaining momentum as a mechanism by which candidate genes underlying speciation can be identified (Pavey *et al*. 2010; Chapman *et al*. 2013; Dunning *et al*. 2016). However, our results clearly show that different assembly and analysis pipelines can produce different outputs, including biological interpretation of the results, and therefore alternative pipelines should be investigated and optimized.

## Supplementary Material

jkad158_Supplementary_Data

## Data Availability

Raw RNA-seq data are available from the NCBI Sequence Read Archive (SRA) under Bioproject PRJNA931793. Assemblies and DE results are available on FigShare (https://doi.org/10.6084/m9.figshare.22261759 and https://doi.org/10.6084/m9.figshare.23578251) or from the authors upon request. All samples were collected using appropriate permits (see Methods) and are consistent with the stipulations of the Nagoya Protocol. Supplemental material available at G3 online.
