# Supplementary material for: Comparative transcriptomics and gene expression divergence associated with homoploid hybrid speciation in Argyranthemum
Source: G3 (Bethesda). 2023 Jul 21;13(10):jkad158. doi: 10.1093/g3journal/jkad158 (PMC10542503; doi:10.1093/g3journal/jkad158)
Supplement: jkad158_Supplementary_Data [file jkad158_supplementary_data.zip › Supplemental_Figures_G3-2023-404364.docx]

**Comparative transcriptomics and gene expression divergence associated with homoploid hybrid speciation in *Argyranthemum***

**SUPPORTING INFORMATION FIGURES**

**
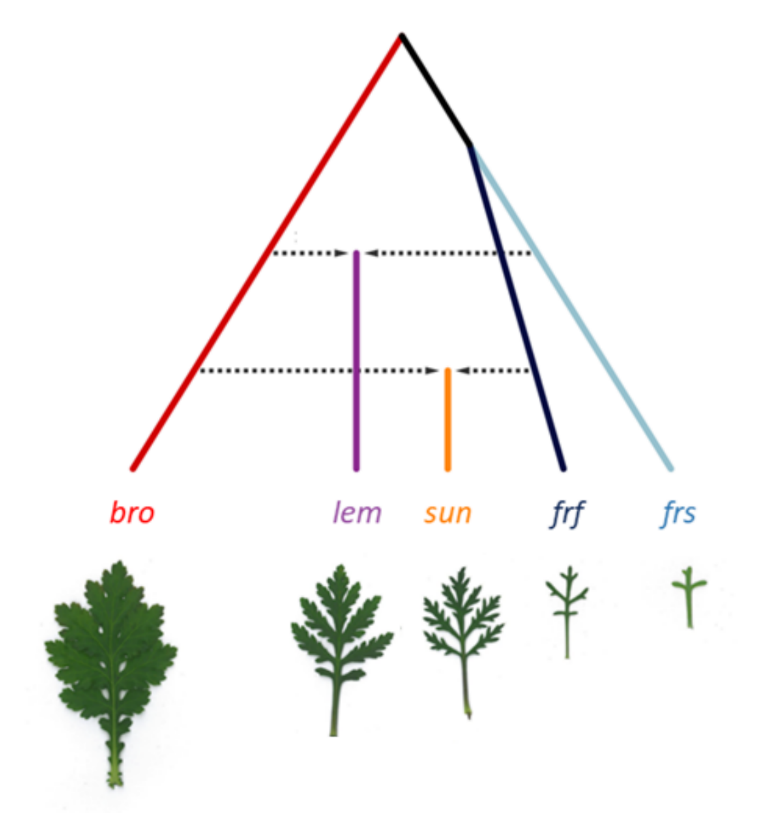
**

Supporting Figure S1 – Schematic diagram of homoploid hybrid speciation in *Argyranthemum* adapted from White *et al.* (2018) showing independent hybrid origins for *A. sundingii* (sun) and *A. lemsii* (lem) from parental taxa *A. broussonetii* (bro), *A. frutescens* subsp. *frutescens* (frf) and *A. frutescens* subsp. *succulentum* (frs).

**
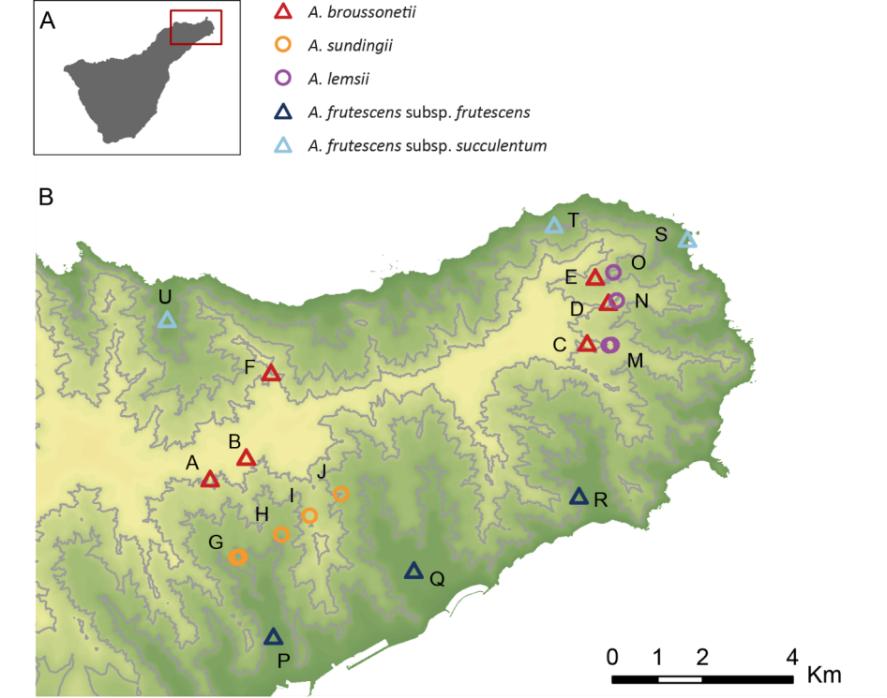
**

Supporting Figure S2 - Populations sampled the Anaga peninsula of Tenerife (see inset). Populations are labelled A-U. Contour lines represent a 200 m change in altitude.


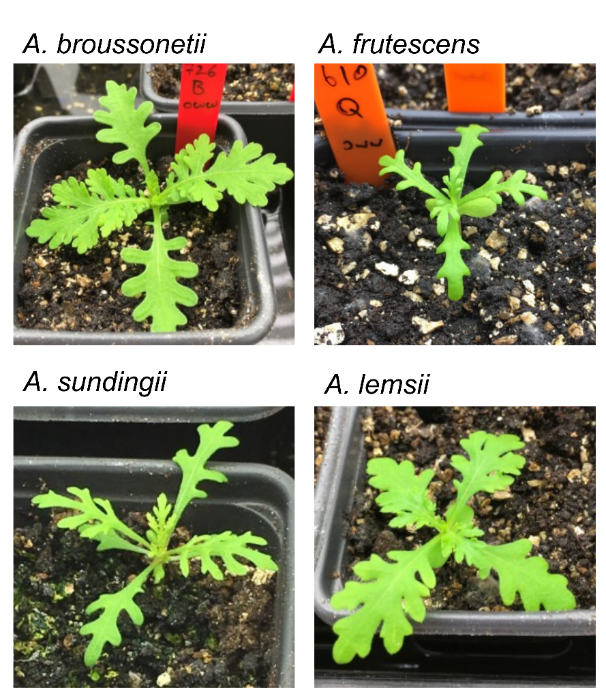


Supporting Figure S3 - Images of seedlings for each taxon showing the morphology at the approximate time of sampling.


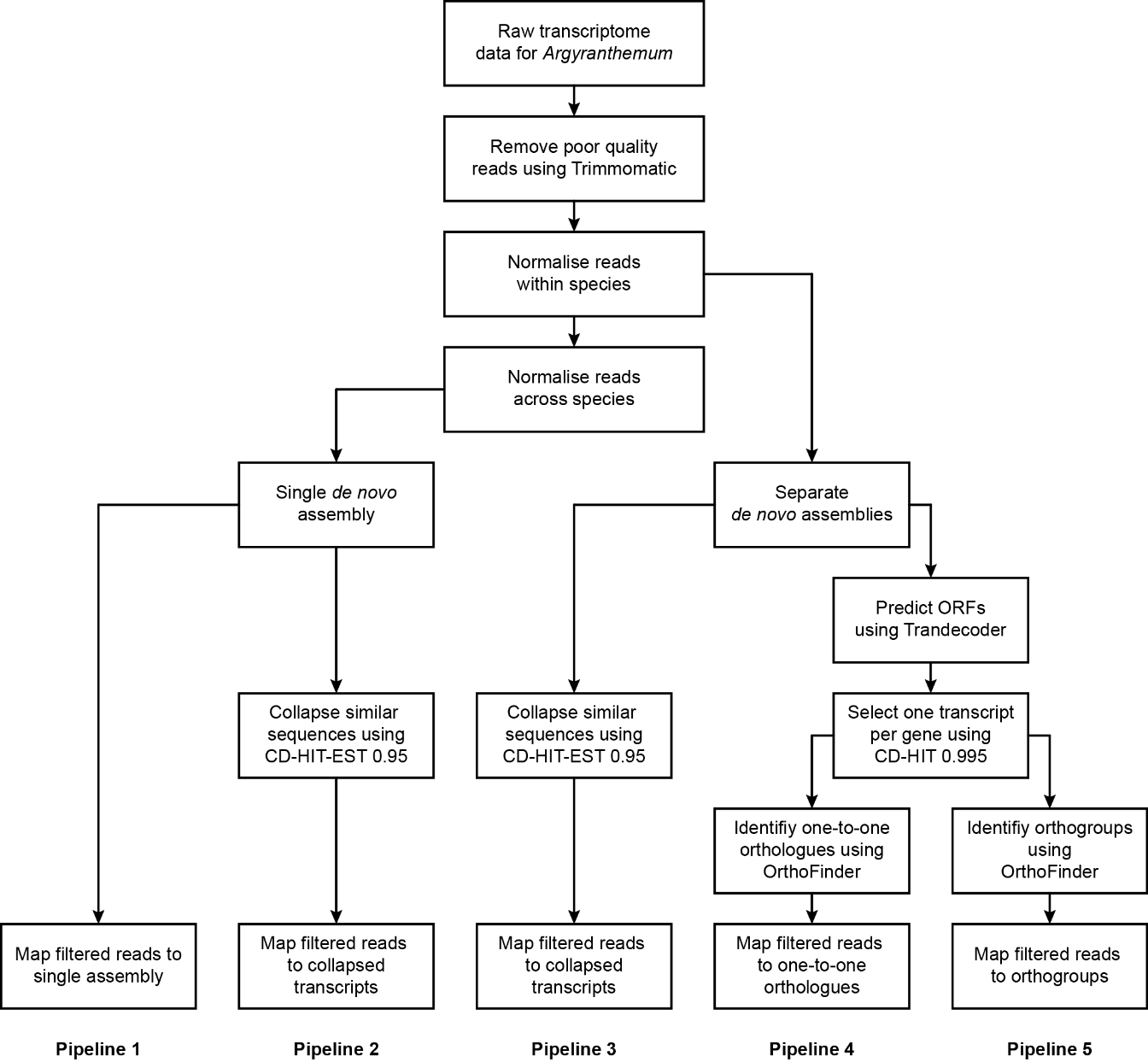


Supporting Figure S4 **-** Schematic of five pipelines used in our transcriptome assembly and transcript quantification. See text for details.


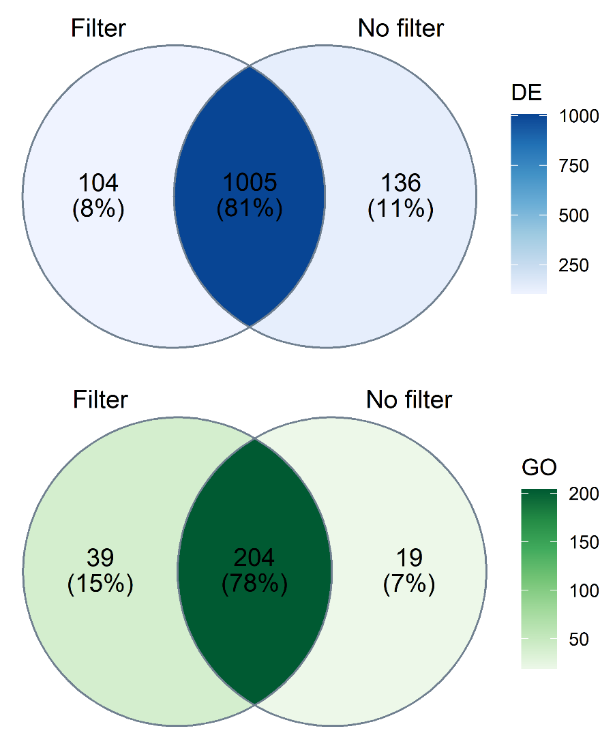


Supporting Figure S5 – Venn diagram depicting the overlap of differentially expressed (DE) loci and enriched Gene Ontology (GO) terms identified for pipeline 5 between *A. broussonetii* and *A. frutescens*, with and without a filter on lowly expressed (< 1TPM) transcripts.


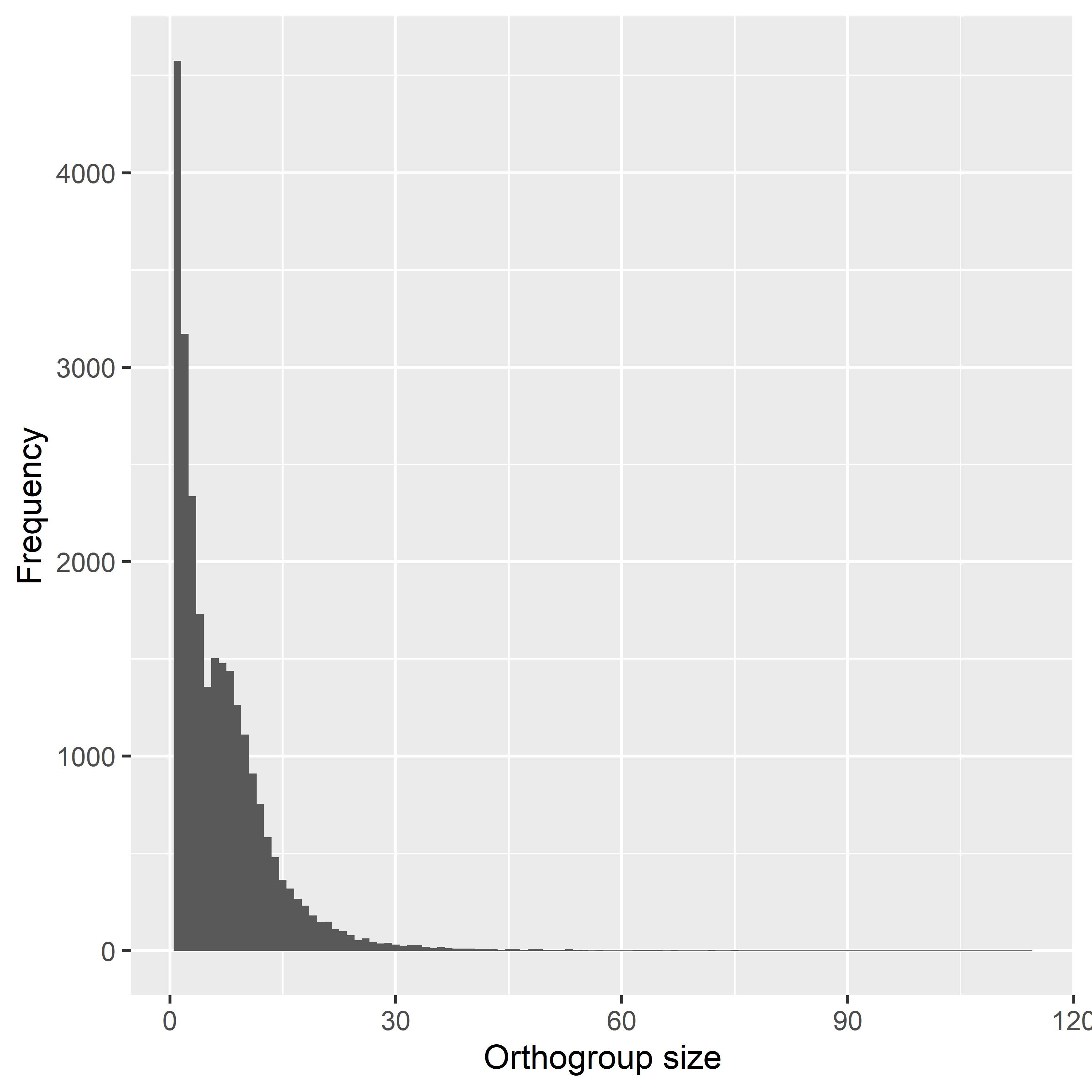


Supporting Figure S6 - Size distribution of orthogroups identified by OrthoFinder for pipeline 5.


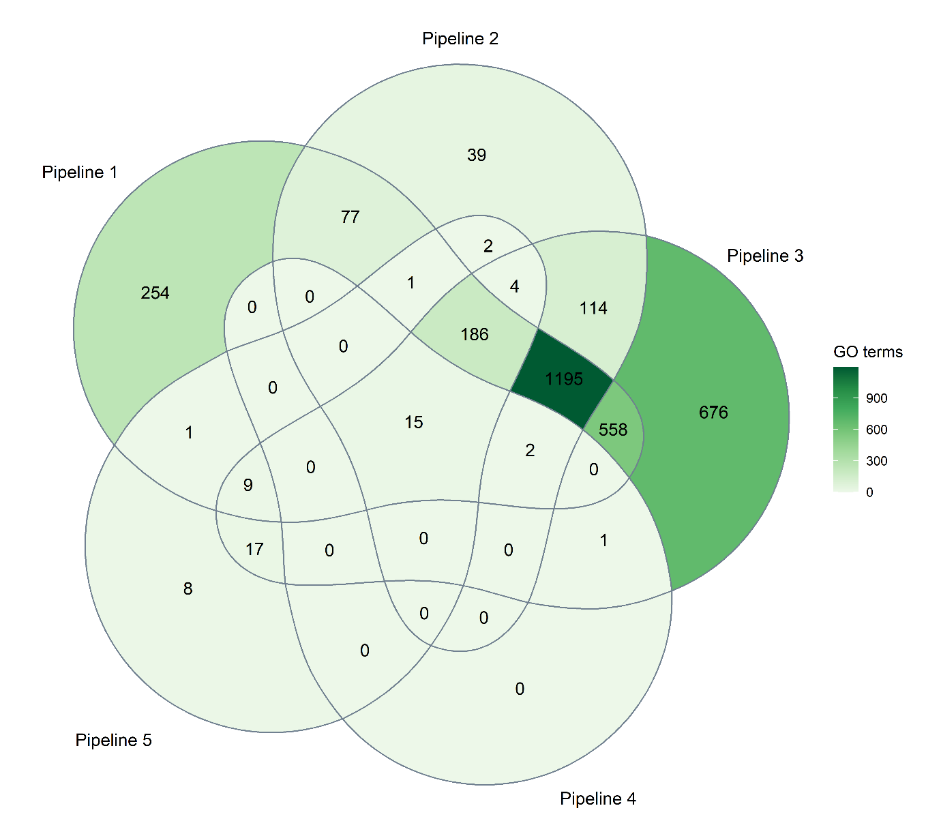


Supporting Figure S7 **-** Venn diagram depicting the number of shared over-represented Gene Ontology (GO) terms for transcripts differentially expressed between the parental species for the five analysis pipelines.
