# Supplementary material for: Comparative transcriptomics and gene expression divergence associated with homoploid hybrid speciation in Argyranthemum
Source: G3 (Bethesda). 2023 Jul 21;13(10):jkad158. doi: 10.1093/g3journal/jkad158 (PMC10542503; doi:10.1093/g3journal/jkad158)
Supplement: jkad158_Supplementary_Data [file jkad158_supplementary_data.zip › Supplemental_Text_G3-2023-404364.docx]

**Comparative transcriptomics and gene expression divergence associated with homoploid hybrid speciation in *Argyranthemum***

## SUPPORTING INFORMATION METHODS

## Quantifying transcript abundance

### Pipeline 1

Transcript abundance for each sample was estimated by mapping filtered (non-normalised) reads to the interspecific assembly using the Trinity script *align_and_estimate_abundance.pl* and the RSEM alignment-based estimation method (Li & Dewey, 2011). Transcript abundance was quantified at the level of Trinity isoforms.

### Pipeline 2

CD-HIT-EST (Fu et al., 2012; Li & Godzik, 2006) was used to collapse potentially orthologous transcripts within the interspecific assembly using a sequence identity threshold of 0.95, to reduce the presence of separately assembled divergent alleles and the problem highlighted in figure 1. Transcript abundance was then quantified as for pipeline 1.

### Pipeline 3

The four species-specific assemblies were combined, and CD-HIT-EST was used in the same way as pipeline 2, to collapse potentially orthologous sequences. Transcript abundance was then quantified as for pipelines 1 and 2.

### Pipeline 4

For each species-specific assembly, peptide open reading frames (ORFs) were predicted for every isoform using Transdecoder version 5.3.0 (<https://github.com/TransDecoder>) and the longest ORF was retained. If no ORF > 100 amino acids were predicted, this isoform was excluded. To minimise the presence of multiple transcripts per gene, CD-HIT was used to cluster peptide sequences within each species using a similarity threshold of 0.995. One-to-one orthologues between species were identified using OrthoFinder version 2.4.0 (Emms & Kelly, 2015) which involves an all-by-all blast search (Camacho et al., 2009) followed by clustering of transcripts using mcl (van Dongen, 2000).

Gene expression was quantified using *align_and_estimate_abundance.pl* using a reference transcriptome comprising a concatenation of the four species-specific transcriptome assemblies and using the *gene_to_trans* flag to specify which transcripts from the four species comprise each one-to-one orthologue. Transcript expression was quantified at the level of the one-to-one orthologues identified.

### Pipeline 5

For the identification of orthogroups we carried out the same steps as for pipeline 4, but we also included representative peptide sequences (primary transcript only) from five reference taxa, i.e. *Helianthus annuus* (Badouin et al., 2017), *Lactuca sativa* L. (Reyes-Chin-Wo et al., 2017), *Solanum lycopersicum* L. (The Tomato Genome Consortium, 2012), *Mimulus guttatus* DC. (Hellsten et al., 2013) and *Arabidopsis thaliana* (Lamesch et al., 2012) in our OrthoFinder analysis to improve orthogroup inference. Peptide sequences for the reference taxa were downloaded from Phytozome. In contrast to pipeline 4, we did not restrict this analysis to the one-to-one orthologues.

From manual inspection, it was clear that there were paralogous transcripts in some of the larger orthogroups (i.e. alignments contained many gaps and transcripts were not monophyletic with respect to genera). To remove likely paralogue-containing orthogroups, we identified and retained only those orthogroups which were monophyletic with respect to genera using the reconciled gene trees generated by OrthoFinder and the R script *check_genus_monophyly.R* (<https://gist.github.com/josephwb/f3d35f8833a07f71002af7726b12652b>; accessed 29/09/2018). Examples of orthogroups removed and selected are presented below as Supporting Information Figures S8 and S9, respectively. After selecting these, expression was quantified in the same way as pipeline 4, with transcripts from the same orthogroup identified as such using the *gene_to_trans* flag, therefore quantifying transcript expression at the level of the orthogroup.


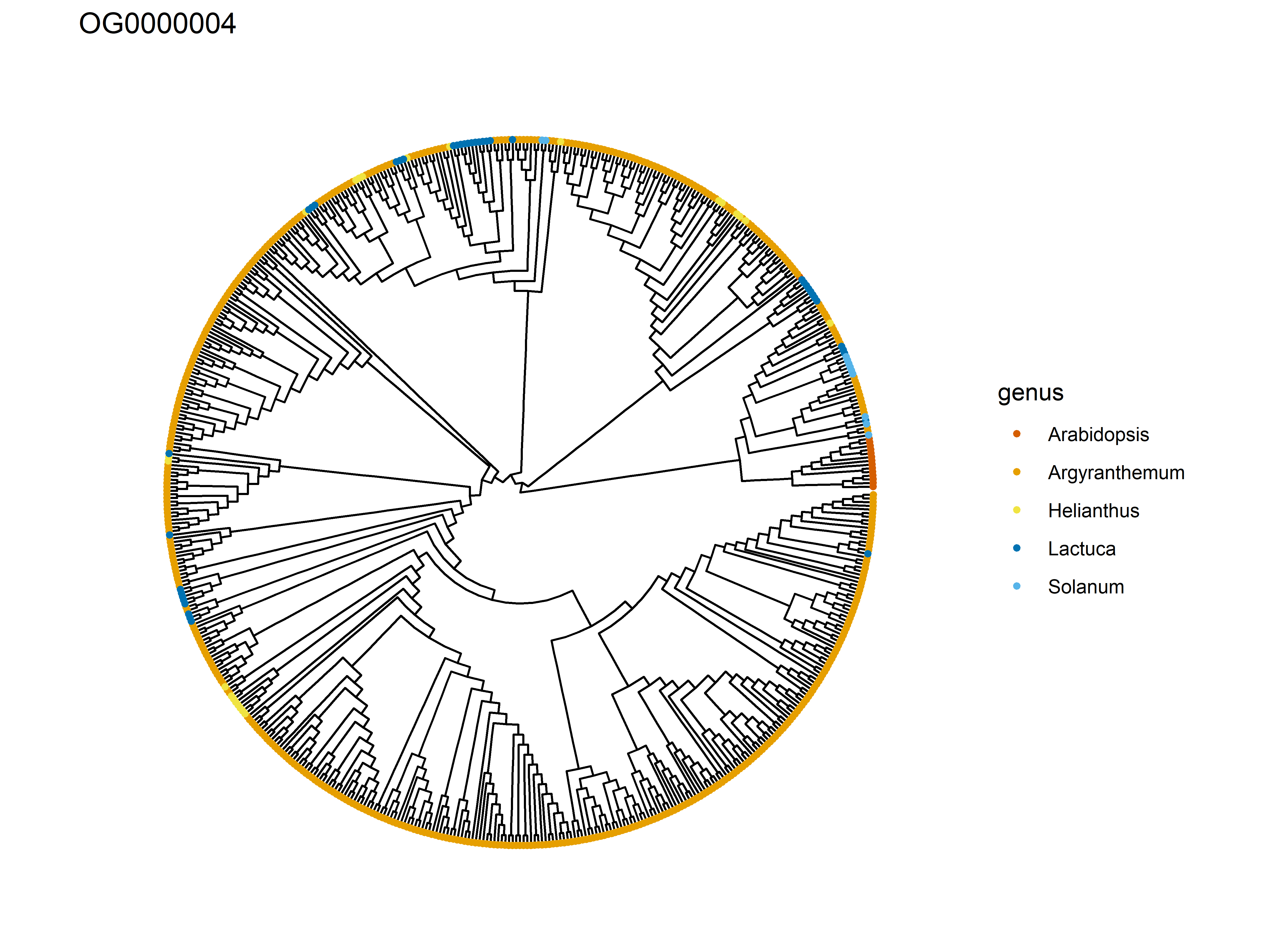


Supporting Figure S8 - Example of likely paralogue-containing orthogroup (OG0000004) removed from our analysis. Note the sequences from each genus are non-monophyletic.


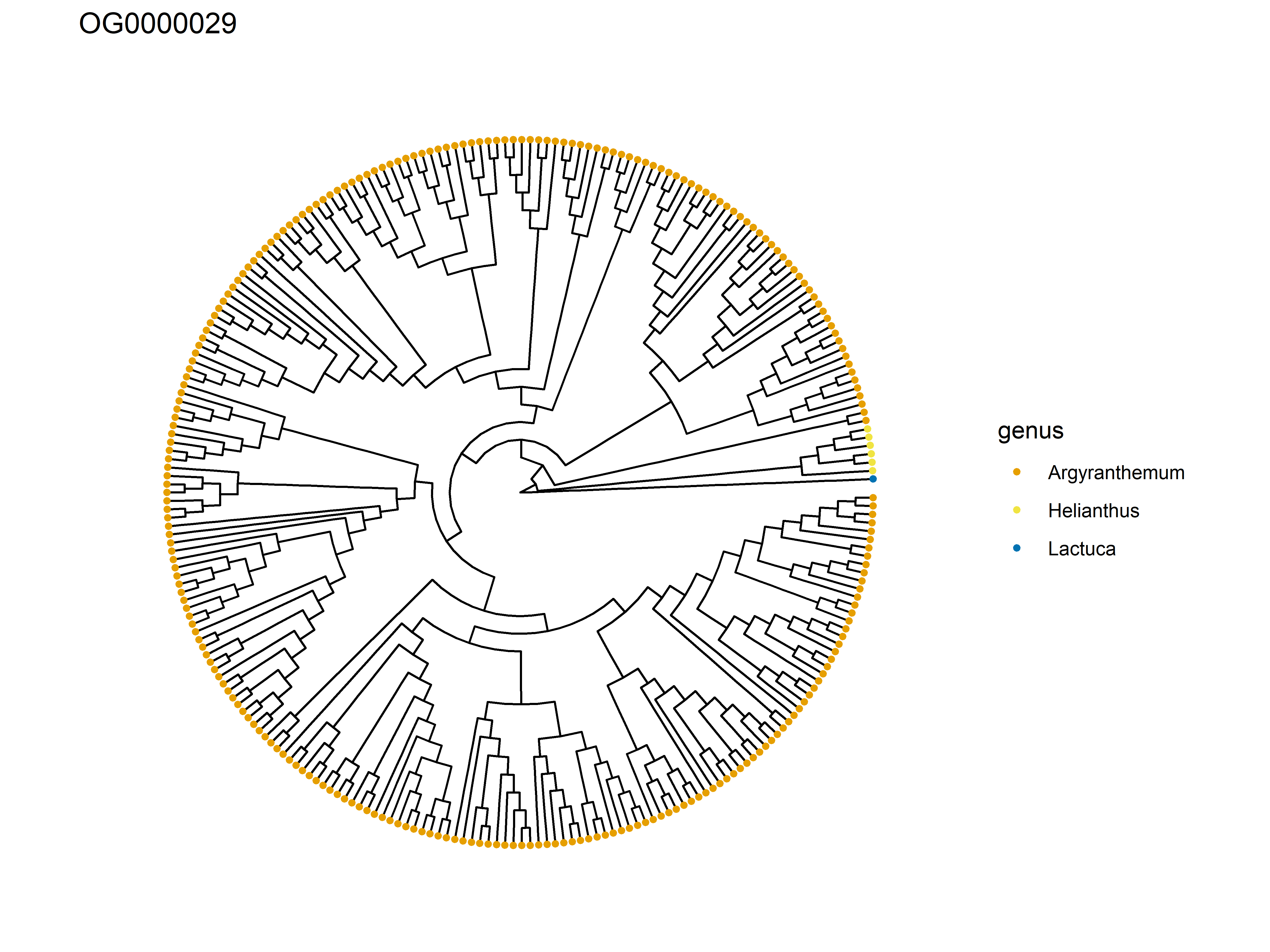


Supporting Figure S9 - Example of likely non-paralogue-containing orthogroup (OG0000029) included in our analysis. Note the sequences from each genus are monophyletic.

## Differential expression

Following transcript quantification, an expression matrix with counts and TMM-normalised counts was built for each pipeline using the Trinity script *abundance_estimates_to_matrix.pl*. Differentially expressed (DE) transcripts, orthologues or orthogroups (hereafter, simply loci) were identified in pairwise comparisons amongst species using edgeR (McCarthy et al., 2012; Robinson et al., 2009) in R (R Core team, 2020) within Trinity, utilising the scripts *run_DE_analysis.pl* and *analyze_diff_expr.pl*. The cut-off for differentially expressed transcripts was a *P* value < 0.05 after false discovery rate (FDR) correction (Benjamini & Hochberg, 1995).

Note that we did not remove lowly expressed loci/orthogroups for any pipeline following the recommendation in the Trinity documentation since this removal could result in the loss of biologically relevant transcripts. Nevertheless, to examine the effect of lowly expressed transcript removal, we carried out a parallel analysis of pipeline 5 but after removing transcripts with low expression (<1 TPM in all samples). This made minor differences to the results with ca. 80% of DE transcripts and over-represented GO terms found in both analyses (Supporting Figure S5). No fold-change cut-off was used (see below).

## Pipeline comparison

To examine whether pipeline choice could result in fundamentally different biological conclusions, we identified Gene Ontology (GO) terms that were over-represented in the lists of loci DE between the parents using TopGO (Alexa & Rahnenfuhrer, 2021) with significantly enriched terms identified using a *P* value < 0.01 with the weight01 algorithm. GO terms were based on the top *Arabidopsis* hit and Fisher’s exact tests (FDR corrected *P* value < 0.05). For pipelines 1-3 we used the top *Arabidopsis* BLAST hit for the locus, and for pipelines 4 and 5 we used the *Arabidopsis* hit with the lowest e-value out of the transcripts present in each orthologue/orthogroup.

To select a pipeline for further analysis, we aimed to determine the extent to which true orthologues were not correctly assembled in each pipeline. If this were the case, we would expect to find that DE transcripts in that pipeline are more likely than expected by chance to have a reciprocal best blast hit (RBBH) which is also DE (i.e., Figure 1; Gene B). To quantify this, we first carried out an all-by-all blast within each of the five assemblies using the longest transcript from orthologues and orthogroups as representatives. We then classified loci into three categories: (1) RBBHs where both of the pair are DE, (2) RBBHs where only one of the pair is DE, and (3) RBBHs where neither of the pair is DE. We then used χ^2^ tests to determine if there was deviation from that expected by chance and a φ correlation to estimate skew in the χ^2^ test.

This first test does not inform us whether the skew is due to orthologues not co-assembling (Figure 1B) or truly DE paralogous loci not co-assembling (Figure 1C). However, these two possibilities can be differentiated by the direction of expression. For the former, the RBBHs are reciprocally DE (Figure 1B), whereas for the latter, the two are DE in the same direction (Figure 1C). We therefore further identified, within the RBBHs where both of the pair are DE (i.e. category 1 above), those which were reciprocally DE and those which were DE in the same direction. We reason that if the pipeline had reduced the problem of orthologues not co-assembling then we would expect less reciprocally DE transcripts than expected by chance (compared to the direction of DE in all RBBHs).

From these two tests, the pipeline with the least evidence of having incorrectly assembled/grouped true orthologues, whilst also optimising the number of loci available for the expression analyses, was used for further analyses of the data to examine HHS.

## SUPPORTING INFORMATION RESULTS

## Pipeline comparison

In all pipelines, χ^2^ tests found that RBBHs where both members are DE occurred more often than expected by chance (all *P* < 0.05; Table 2), the pattern that could arise if false patterns of DE were being recovered because orthologous loci were not co-assembling (Figure 1), although this was only marginally significant for pipeline 4 (P = 0.0108).

The number of reads mapping back to the same species’ transcriptome was greater than mapped to another species (Supporting Information Figure S10), presumably, at least in part, due to sequence divergence which could prevent some orthologous loci co-assembling.


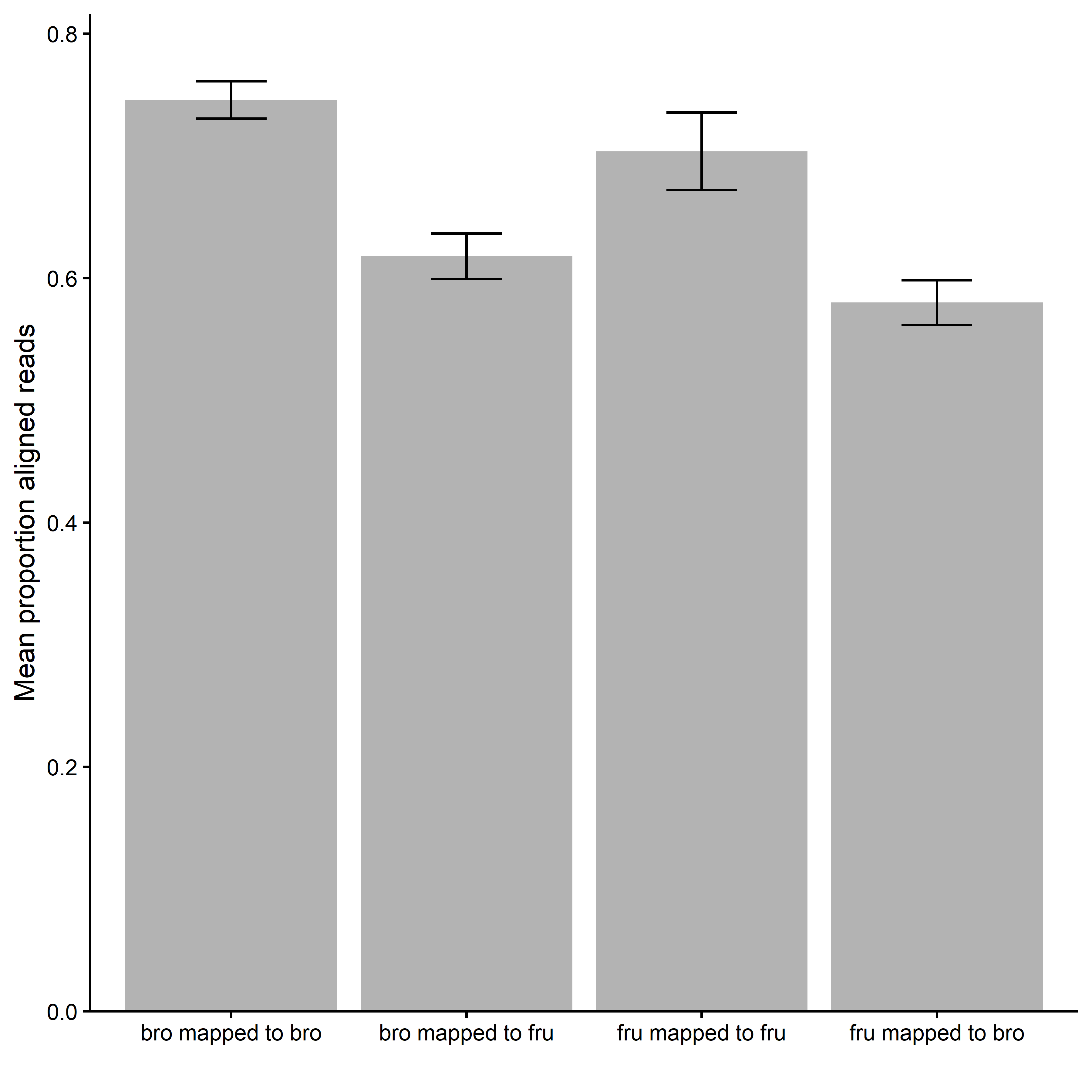


Supporting Figure S10 - Mean proportion of mapped reads with error bars for standard deviation for bro (A. broussonetii) mapped against bro, bro mapped against fru (A. frutescens), fru mapped against fru and fru mapped against bro.

We also suggest above that some skew might be expected if two (relatively divergent and therefore non co-assembling) gene paralogues are genuinely DE (Figure 1C); as might be expected if the paralogues perform a similar function. To investigate this, we examined the direction of DE (i.e., bro > fru or bro < fru) in all pairs of RBBHs where both members are DE. We then compared this to the observed numbers of reciprocally DE and same direction DE in this subset of RBBHs.

Excluding pipeline 4 (see below), very similar number of bro > fru and bro < fru transcripts were present, resulting in an almost 50:50 expectation of reciprocally and same direction DE RBBHs (i.e., orthologues not co-assembling vs. paralogues not co-assembling, respectively). For pipelines 1-3, the observed percentage of reciprocally DE loci was similar to this, varying between 40.3% and 56.6%, whereas for pipeline 5 the observed percentage was only 19.4% (Table 2). This suggests that for pipeline 5 we had reduced the number of putative non-co-assembling orthologues. For pipeline 4 only four RBBHs (eight loci) were both DE, with a skew in the number of bro > fru vs. bro < fru transcripts present (7 vs 1), resulting in an estimation of 22% of pairs being reciprocally DE, which matched the observed result (one out of the four; i.e. 25%).

Considering the evidence above we employed pipeline 5 for further downstream analyses as it appears to reduce the problem of orthologous loci not co-assembling (seen in pipelines 1-3) and does not remove a large proportion of loci prior to analysis (seen in pipeline 4).

**SUPPORTING INFORMATION REFERENCES**

Alexa, A., & Rahnenfuhrer, J. (2021). topGO: Enrichment Analysis for Gene Ontology (R package version 2.40.0).

Badouin, H., Gouzy, J., Grassa, C. J., Murat, F., Staton, S. E., Cottret, L., Lelandais-Brière, C., Owens, G. L., Carrère, S., Mayjonade, B., Legrand, L., Gill, N., Kane, N. C., Bowers, J. E., Hubner, S., Bellec, A., Bérard, A., Bergès, H., Blanchet, N., … Langlade, N. B. (2017). The sunflower genome provides insights into oil metabolism, flowering and Asterid evolution. Nature, 546(7656), 148–152. <https://doi.org/10.1038/nature22380>

Benjamini, Y., & Hochberg, Y. (1995). Controlling the False Discovery Rate: a practical and powerful approach to multiple testing. Journal of the Royal Statistical Society, 57(1), 289–300.

Camacho, C., Coulouris, G., Avagyan, V., Ma, N., Papadopoulos, J., Bealer, K., & Madden, T. L. (2009). BLAST+: Architecture and applications. BMC Bioinformatics, 10, 1–9. <https://doi.org/10.1186/1471-2105-10-421>

Emms, D. M., & Kelly, S. (2015). OrthoFinder: solving fundamental biases in whole genome comparisons dramatically improves orthogroup inference accuracy. Genome Biology, 16(1), 157. <https://doi.org/10.1186/s13059-015-0721-2>

Fu, L., Niu, B., Zhu, Z., Wu, S., & Li, W. (2012). CD-HIT: Accelerated for clustering the next-generation sequencing data. Bioinformatics, 28(23), 3150–3152. <https://doi.org/10.1093/bioinformatics/bts565>

Hellsten, U., Wright, K. M., Jenkins, J., Shu, S., Yuan, Y., Wessler, S. R., Schmutz, J., Willis, J. H., & Rokhsar, D. S. (2013). Fine-scale variation in meiotic recombination in *Mimulus* inferred from population shotgun sequencing. Proceedings of the National Academy of Sciences, 110(48), 19478–19482. <https://doi.org/10.1073/pnas.1319032110>

Lamesch, P., Berardini, T. Z., Li, D., Swarbreck, D., Wilks, C., Sasidharan, R., Muller, R., Dreher, K., Alexander, D. L., Garcia-Hernandez, M., Karthikeyan, A. S., Lee, C. H., Nelson, W. D., Ploetz, L., Singh, S., Wensel, A., & Huala, E. (2012). The Arabidopsis Information Resource (TAIR): Improved gene annotation and new tools. Nucleic Acids Research, 40(D1), 1202–1210. <https://doi.org/10.1093/nar/gkr1090>

Li, B., & Dewey, C. N. (2011). RSEM: Accurate transcript quantification from RNA-Seq data with or without a reference genome. *BMC Bioinformatics*, *12*, 323. https://doi.org/10.1186/1471-2105-12-323

Li, W., & Godzik, A. (2006). Cd-hit: A fast program for clustering and comparing large sets of protein or nucleotide sequences. *Bioinformatics*, *22*(13), 1658–1659. <https://doi.org/10.1093/bioinformatics/btl158>

McCarthy, D. J., Chen, Y., & Smyth, G. K. (2012). Differential expression analysis of multifactor RNA-Seq experiments with respect to biological variation. Nucleic Acids Research, 40(10), 4288–4297. <https://doi.org/10.1093/nar/gks042>

R Core team. (2020). R: A language and environment for statistical computing (3.6.3). R Foundation for Statistical Computing.

Reyes-Chin-Wo, S., Wang, Z., Yang, X., Kozik, A., Arikit, S., Song, C., Xia, L., Froenicke, L., Lavelle, D. O., Truco, M. J., Xia, R., Zhu, S., Xu, C., Xu, H., Xu, X., Cox, K., Korf, I., Meyers, B. C., & Michelmore, R. W. (2017). Genome assembly with in vitro proximity ligation data and whole-genome triplication in lettuce. Nature Communications, 8, 14953. <https://doi.org/10.1038/ncomms14953>

Robinson, M. D., McCarthy, D. J., & Smyth, G. K. (2009). edgeR: A Bioconductor package for differential expression analysis of digital gene expression data. Bioinformatics, 26(1), 139–140. <https://doi.org/10.1093/bioinformatics/btp616>

The Tomato Genome Consortium. (2012). The tomato genome sequence provides insights into fleshy fruit evolution. Nature, 485(7400), 635–641. <https://doi.org/10.1038/nature11119>

van Dongen, S. M. (2000). Graph Clustering by Flow Simulation (Doctoral dissertation). University of Utrecht.
